# Structural and Functional Projections of the Nucleus Basalis of Meynert and Their Changes After Cognitive Training in Individuals With Mild Cognitive Impairment

**DOI:** 10.1111/cns.70194

**Published:** 2024-12-26

**Authors:** Qingzheng Lu, Yu Wang, Bingqian Qu, Caixia Wang, Xiao Su, Siqi Wang, Yi Xing, Wen Qin, Yi Tang, Nan Zhang

**Affiliations:** ^1^ Department of Neurology, Tianjin Neurological Institute Tianjin Medical University General Hospital Tianjin China; ^2^ Department of Neurology Tianjin Medical University General Hospital Airport Site Tianjin China; ^3^ Department of Neurology Baotou Central Hospital Baotou China; ^4^ Department of Neurology and Innovation Center for Neurological Disorders, Xuanwu Hospital, National Center for Neurological Disorders Capital Medical University Beijing China; ^5^ Department of Radiology and Tianjin Key Laboratory of Functional Imaging Tianjin Medical University General Hospital Tianjin China

**Keywords:** computerized cognitive training, diffusion tensor imaging, mild cognitive impairment, nucleus basalis of Meynert, resting‐state fMRI

## Abstract

**Aims:**

The nucleus basalis of Meynert (NBM) is a major source of cholinergic innervation in the central nervous system. We aimed to investigate the characteristics of structural and functional alterations in the NBM and its projections in patients with mild cognitive impairment (MCI) and the effects of computerized cognitive training (CCT).

**Methods:**

Forty‐five patients with MCI and 45 cognitively unimpaired controls (CUCs) were recruited. NBM volume, mean diffusivity (MD) of NBM white matter (WM) projections, and functional connectivity (FC) of projections of the NBM were measured with T1‐weighted imaging, diffusion tensor imaging (DTI), and resting‐state functional magnetic resonance imaging (rs‐fMRI). Thirty‐six MCI patients were randomly assigned to receive CCT or control training. The effects of CCT on the neuropsychological measures and MRI properties were analyzed with a linear mixed model (LMM).

**Results:**

We detected that compared with the CUCs, the MCI patients had a reduced volume of the NBM and a greater MD of both cholinergic pathways. Increased MD values of both pathways were related to lower scores of global cognition, processing speed and attention in all participants. After CCT intervention, significant group × timepoint effects on score of the Backward Digit Span and the FC between NBM and right putamen were observed in the CCT group compared to the control group.

**Conclusion:**

NBM atrophy and WM pathway disruption occurred in MCI patients and were correlated with cognitive impairment. Working memory and the FC between NBM and right putamen could be improved by cognitive training.

## Graphical abstract

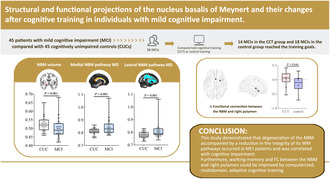

## 1 Background

The large population of patients with dementia has increasingly imposed a heavy burden on the public and healthcare systems [1]. Mild cognitive impairment (MCI) is a stage in which cognitive decline exceeds what is expected for an individual's age and level of education but does not significantly affect activities of daily living [2]; MCI has a prevalence of 10%–20% in adults aged ≥ 65 years [3]. Although some patients with MCI can stabilize or return to normal over time, more than half develop dementia within 5 years, particularly Alzheimer's disease (AD) [2]. Since pathological injuries in the brains of patients with AD dementia are usually irreversible, it is vital to understand the underlying neurobiological changes and identify effective intervention targets for MCI.

The pathogenesis and pathophysiological process of AD are complex [4]. The cholinergic hypothesis suggests that neurocholinergic neuronal degeneration or disruption of the brain's cholinergic system is one of the main neurotransmitter‐based pathogeneses of AD [5]. The nucleus basalis of Meynert (NBM), a major source of cholinergic innervation in the cerebral cortex, has been found to have severe cholinergic neuronal deficits and might degenerate in the early stages of AD [6].

Furthermore, cholinergic projections from the NBM to the cortex are composed of separate and organized bundles. Two main cholinergic fiber bundles have been identified: a medial pathway that passes through the cingulum toward the cingulate, retrosplenial, and subcallosal cortices and a lateral pathway that passes through the external capsule and uncinate fasciculus to innervate the insula as well as the frontal, parietal, and temporal cortices [7]. Disruption of cholinergic white matter (WM) pathway integrity has been associated with AD‐related pathology [8].

Recent magnetic resonance imaging (MRI) studies have explored in vivo NBM cholinergic pathway changes in MCI patients, including NBM volume reduction [9], increased mean diffusivity (MD) of NBM WM projections measured with diffusion tensor imaging (DTI) [8], and reduced functional connectivity (FC) based on resting‐state functional MRI (rs‐fMRI) between the NBM and brain regions receiving cholinergic projections [10]. The regions with functional dysconnectivity do not spatially overlap with those with structural degeneration [11]. In addition, most of the above studies adopted a single‐modality path, that is, structural or functional, which limits the interpretability of the brain‐to‐performance correspondence [12].

Computerized cognitive training (CCT) has been recommended as a safe and productive approach for neurocognitive improvement in both healthy elderly individuals and patients with early stages of cognitive impairment, for example, MCI [13]. The potential mechanism underlying CCT is that effective cognitive exercises might promote or rebuild neural reserves, increasing resilience against pathological degeneration [14]. Several beneficial effects have been observed after CCT, such as neurochemical activation [15], altered fluorodeoxyglucose uptake [16], and decreased β‐amyloid burden [17]. However, whether the volume and structural or functional projections of the NBM are influenced by CCT is still unknown.

Based on the previous findings, we hypothesized that changes in the NBM and its related projections could occur in MCI patients and that these changes could be alleviated by CCT. In this study, we explored changes in NBM volume and the NBM cholinergic pathway in patients with MCI using multimodal MRI data. We further observed the effects of a 12‐week CCT regimen on cognitive and NBM pathway improvements.

## 2 Methods

### 2.1 Participants

This study was approved by the Ethics Committee of Tianjin Medical University General Hospital. Forty‐five patients with MCI and 45 age‐ and sex‐matched cognitively unimpaired controls (CUCs) were recruited from Tianjin Medical University General Hospital; the participants provided written informed consent and underwent comprehensive clinical assessments, including demographics and medical history collection, neuropsychological assessments, and brain MRI.

The inclusion criteria for MCI patients were as follows: (1) met the MCI diagnostic criteria as defined by Petersen [18]; (2) were aged 50–85 years; (3) had a Clinical Dementia Rating (CDR) [19] score = 0.5 and a Mini‐Mental State Examination (MMSE) [20] score ≥ 24; and (4) were able to communicate proficiently in Chinese. The exclusion criteria were as follows: (1) a diagnosis of a major neurocognitive disorder (synonymous with dementia) according to the DSM‐5 [21]; (2) cognitive impairment caused by cerebrovascular disease (stroke history or significant vascular lesions on MRI, such as lacunar infarcts, and moderate to severe WM hyperintensities with a Fazekas score > 2) or other definite neurological, mental, or systemic disorders, such as multiple sclerosis, hydrocephalus, severe depression, alcoholism, drug addiction, thyroid dysfunction, vitamin B12 deficiency, HIV infection, and neurosyphilis; (3) severe aphasia or physical disability that hinders completion of the neuropsychological examination; and (4) inability to undergo an MRI scan.

CUCs met the following inclusion criteria: (1) aged 50–85 years; (2) no reports of self‐perceived cognitive decline and normal performance on objective neuropsychological assessments; (3) CDR score = 0 and MMSE score ≥ 26; (4) no abnormal findings such as significant cerebrovascular lesions, tumors, hydrocephalus, or significant global or regional atrophy on MRI; and (5) no medical history of neurological or psychiatric disorders that might influence cognitive performance.

### 2.2 Neuropsychological Testing

In addition to the Chinese version of the MMSE and the Montreal Cognitive Assessment (MoCA) [22], which assessed global cognitive function, the Trail Making Test A (TMT‐A) and the Digit Span (DS) test were also performed. The TMT‐A was used to evaluate visual scanning and visuomotor processing speed [23]; during this assessment, participants were asked to connect randomly arranged numbers from 1 to 25 following the number sequence as quickly as possible. Because the distributions of the TMT‐A scores were strongly skewed, the data were log transformed for analysis. In addition, the TMT‐A score was multiplied by −1 to be consistent with other tests, with a higher score indicating better performance. For the DS test, participants were required to recall a series of numbers in the order they were presented (forward) and in the reverse order (backward) [24]. The forward DS test was used as an indicator of storage and maintenance memory, and the backward DS test was used as an indicator of working memory capacity, which processes and renews the information stored in short‐term memory [25].

### 2.3 MRI Acquisition

All participants underwent a multimodal MRI scan on a 3.0‐Tesla MRI scanner (Discovery MR750, General Electric, Milwaukee, WI, USA). High‐resolution structural T1‐weighted images were acquired with the following parameters: repetition time (TR) = 8.14 ms; echo time (TE) = 3.17 ms; field of view (FOV) = 256 mm × 256 mm; flip angle (FA) = 12°; slice thickness = 1 mm; and 188 sagittal slices. The parameters of the rs‐fMRI sequence were as follows: TR = 2000 ms; TE = 30 ms; matrix = 64 × 64; FOV = 220 × 220 mm; FA = 90°; slice thickness = 3 mm; and number of slices = 36. DTI images were obtained using an echo–planar imaging (EPI) sequence with the following parameters: TR = 6000 ms; TE = 63.2 ms; 64 diffusion‐weighted directions with a b value of 1000 s/mm^2^; flip angle = 90°; FOV = 256 × 256 mm; and 50 contiguous 6‐mm‐thick axial slices.

### 2.4 Voxel‐Based Morphometric Analysis of NBM Volume

The NBM volume was determined from the T1‐weighted MR images by the CAT12 toolbox [26] implemented in SPM12 (http://www.fil.ion.ucl.ac.uk/spm/software/spm12/). First, all the images were skull‐stripped and gray matter (GM)‐segmented. Then, spatial registration to the Montreal Neurological Institute (MNI) 152 standard space was performed. After preprocessing, individual NBM volumes were calculated by summing the GM voxels within the NBM mask and controlling for the total intracranial volume (TIV) to account for intersubject variability in brain size [27].

### 2.5 NBM Cholinergic WM Tracts Based on Diffusion MRI

The DTI data were processed using the FSL toolbox (version 6.0.6, FMRIB, Oxford, UK) according to the procedure (Figure S1) described in a previous study [9]. First, the nonbrain tissues were removed [28], and eddy currents and head motion were corrected [29]. FSL's dtifit was then applied to fit a diffusion tensor model to the data [30]. The diffusion parameters in the standard ball‐and‐stick model [31] for each voxel were estimated using the algorithm implemented in FSL (BedpostX), assuming three fibers modeled per voxel.

The two main WM pathways originating from the NBM and passing through the cingulum and external capsule were identified [7]. The NBM region of interest (ROI) was based on a cytoarchitectonic map of the cholinergic basal forebrain (CBF) in the MNI T1 space [32]. The cingulum and external capsule masks were obtained from Johns Hopkins University (JHU) ICBM‐DTI‐81 WM Labels in the FSL [33]. In addition, the anterior commissure and brainstem ROIs, which were obtained from the Harvard–Oxford subcortical structural atlas, were used as exclusion masks to avoid mixing fiber tracts from noncholinergic pathways. These ROI masks were registered into each subject's individual space by inversely applying the registration parameters.

Probabilistic tracking was performed by generating 5000 random samples from the NBM ROI voxels and propagating them through a local probability density function that estimated the diffusion parameters [31]. In accordance with a previously described procedure [8], our study selected the tracts from all the CUCs to create a group tract mask for the medial and lateral pathways and included only the voxels that were present in at least 60% of the participants. This threshold was chosen based on visual inspection of the resulting group tracts. Then, the MD of the medial and lateral group tracts was extracted for each participant.

### 2.6 FC Projections of the NBM Based on rs‐fMRI

The rs‐fMRI data for FC analysis were obtained from a 6‐min recording and consisted of 180 scans. The preprocessing was performed using the Data Processing and Analysis of Brain Imaging (DPABI, http://rfmri.org/DPABI) toolbox based on SPM12. To ensure steady signals, the initial 10 volumes of the rs‐fMRI images were discarded. Other preprocessing steps included slice timing, realignment, spatial normalization, bandpass temporal filtering (0.01–0.1 Hz), and nuisance regression. Finally, spatial smoothing was performed with a full width at half‐maximum (FWHM) kernel of 6 mm.

The CBF can be subdivided into an anterior‐medial cluster (aCBF) and a posterior‐lateral cluster (pCBF), the latter of which covers most of the NBM [34]. We performed FC analyses between the NBM and three correlated brain regions, including the bilateral putamen and right amygdala, which are referred to as the FC maxima of pCBF, to represent the interconnections within the NBM functional projections. For individual subjects, we calculated Pearson's correlation coefficients between the time course of the NBM and the three correlated brain regions. The Fisher's *Z* transformation correlation coefficients were then extracted.

### 2.7 Cognitive Training Intervention

Thirty‐six MCI patients participated in a further CCT study with a single‐blind randomized controlled design, which was approved by the Ethics Committee of the General Hospital of Tianjin Medical University. In addition to the inclusion criteria for the main study, MCI patients who participated in the CCT study were not eligible if they used medications associated with cognitive enhancement or were participating in other therapeutic clinical trials. These participants provided written informed consent and learned how to perform cognitive training on a tablet computer.

The participants were randomized into CCT and control groups via an interactive web system. An adaptive training program for multiple domains, including attention, working memory, long‐term memory, processing speed, and reasoning, was used in the intervention group. Participants needed to complete 40 min of training per day (five 8‐min tasks) at least 4 days per week. Within each task, 80% or greater accuracy was required to advance to the next difficulty level. Patients in the control group performed tasks of perception and reaction speed with the same training intensity as the intervention group. However, the content and difficulty of the training tasks in the control group were fixed and were not adjusted according to the patient's performance.

All participants completed their training tasks using tablet computers at home and were supervised by researchers over the internet (www.66nao.com) to guarantee the fulfillment of the training. Neuropsychological and MRI assessments were conducted at baseline (reference to data from the main study) and within 3 months after completing the 12‐week cognitive training. We focused on the effects of CCT on NBM structure and function, including the NBM volume, the MD of NBM WM projections and the FC between the NBM and three related brain regions.

### 2.8 Statistical Analysis

Statistical analyses were mainly performed with SPSS version 26.0, and the figures were produced via GraphPad Prism version 9.3.0. The Kolmogorov–Smirnov test was used to examine the normality of distributions for all continuous variables, and the Wilcoxon rank‐sum test was applied to nonnormally distributed variables. Square roots were taken for the NBM volume as well as for the MD values of the cholinergic pathways and the remaining WM control mask to ensure that these data satisfied a normal distribution. Levene's test was used to verify the homogeneity of variance for normally distributed data, and Welch's analysis of covariance (ANCOVA) was performed for variables with heterogeneous variance via the R statistical software (version 4.3.3). The chi‐squared test was applied to categorical variables. The results were deemed statistically significant at two‐tailed *p* < 0.05.

Baseline demographics and neuropsychological test results were compared between the MCI and CUC groups as well as between the CCT and control groups involved in cognitive training. The Wilcoxon rank‐sum test was applied to all variables, except for sex, which was analyzed using the chi‐squared test.

MRI parameters were first analyzed in all MCI patients and CUCs. To compare between‐group differences in the extracted NBM volumes, one‐way ANCOVA was performed, setting the group as an independent variable and age, sex, education, and TIV as covariates. MD of the two NBM tracts and the remaining WM control mask were compared between the groups with a univariate ANCOVA controlling for age, sex, and education. For functional MRI, we analyzed groupwise differences in FC between the NBM ROI and three related brain regions (bilateral putamen and right amygdala) using ANCOVA with age, sex, and education as covariates. To explore the potential neural mechanism involving the NBM and underlying cognitive impairment, Spearman's correlations were calculated between the neuropsychological scores and MRI metrics for all participants. We also performed correlation analyses between MRI metrics to evaluate their interrelationships.

We then examined the effect of the CCT program on cognitive function and the NBM in MCI patients. The Group × Timepoint interaction effects on the neuropsychological measures and MRI properties were tested with a linear mixed model (LMM) for repeated measures at baseline and at the end of the intervention. Time was assigned as the repeated variable. Group, time, and group‐by‐time were included as fixed effects. For comparisons of FC, GRETNA software was used to construct a visible brain graph.

## 3 Results

### 3.1 Demographics and Cognitive Performance of All Participants

The demographic data and clinical information of all participants at baseline are shown in Table 1. There were no significant differences in age, sex, or years of education between the CUC group and the MCI group. The MMSE (*W* = 626, *p* = 0.002), MoCA (*W* = 425, *p* < 0.001), forward DS test (*W* = 658, *p* = 0.003), and backward DS test (*W* = 751, *p* = 0.027) scores were worse in the MCI group than in the CUC group.

**TABLE 1:** Demographics, neuropsychological assessments, and NBM parameters of all participants.

	CUC (*n* = 45)	MCI (*n* = 45)	*W*/χ^2^/*F*	*p*
Age, years	67 [65, 70]	69 [66, 73]	1235	0.073
Years of education	12 [9, 15]	12 [9, 16]	969	0.716
Sex, M/F	16/29	12/33	0.829	0.362
MMSE	0.15 [−0.68, 0.98]	−1.50 [−2.33, 0.15]	626	0.002
MoCA	0.02 [−0.82, 0.85]	−1.65 [−2.48, −0.4]	425	< 0.001
TMT‐A	0.13 [−0.30, 0.66]	−0.25 [−1.40, 0.47]	776	0.057
Forward DS	−0.21 [−0.21, 0.85]	−0.21 [−2.34, −0.21]	658	0.003
Backward DS	0.31 [−0.51, 0.31]	−0.51 [−1.34, 0.31]	751	0.027
NBM volume, mL	0.53 (0.05)	0.50 (0.05)	12.717	< 0.001
MD in the medial NBM pathway^a^, ^b^	0.8110 (0.025)	0.8453 (0.058)	11.594	0.001
MD in the lateral NBM pathway^a^, ^b^	0.7797 (0.024)	0.8107 (0.055)	13.055	0.001
MD in the remaining white matter^b^	0.9826 (0.065)	1.0364 (0.082)	13.654	< 0.001
FC between NBM and left putamen	0.3840 (0.256)	0.4262 (0.232)	0.862	0.356
FC between NBM and right putamen	0.4424 (0.277)	0.4400 (0.283)	0.054	0.817
FC between NBM and right amygdala	0.3513 (0.283)	0.3667 (0.272)	0.414	0.522

*Note:* The normally distributed continuous variables are presented as the mean (standard deviation), and otherwise as the medians [Q1, Q3]. The neuropsychological assessment values are presented as the medians [Q1, Q3] of the *Z* scores.

Abbreviations: CUC, cognitively unimpaired control; DS, Digit Span; FC, functional connection; MCI, mild cognitive impairment; MD, mean diffusivity; MMSE, Mini‐Mental State Examination; MoCA, Montreal Cognitive Assessment; NBM, nucleus basalis of Meynert; TIV, total intracranial volume; TMT, Trial Making Test.

^a^ MD values in the cholinergic pathways were compared via Welch's ANOVA.

^b^ MD values in the cholinergic pathways or the remaining white matter were multiplied by 1000.

### 3.2 Structural and Functional Measures of the NBM and Related Pathways

**FIGURE 1:**
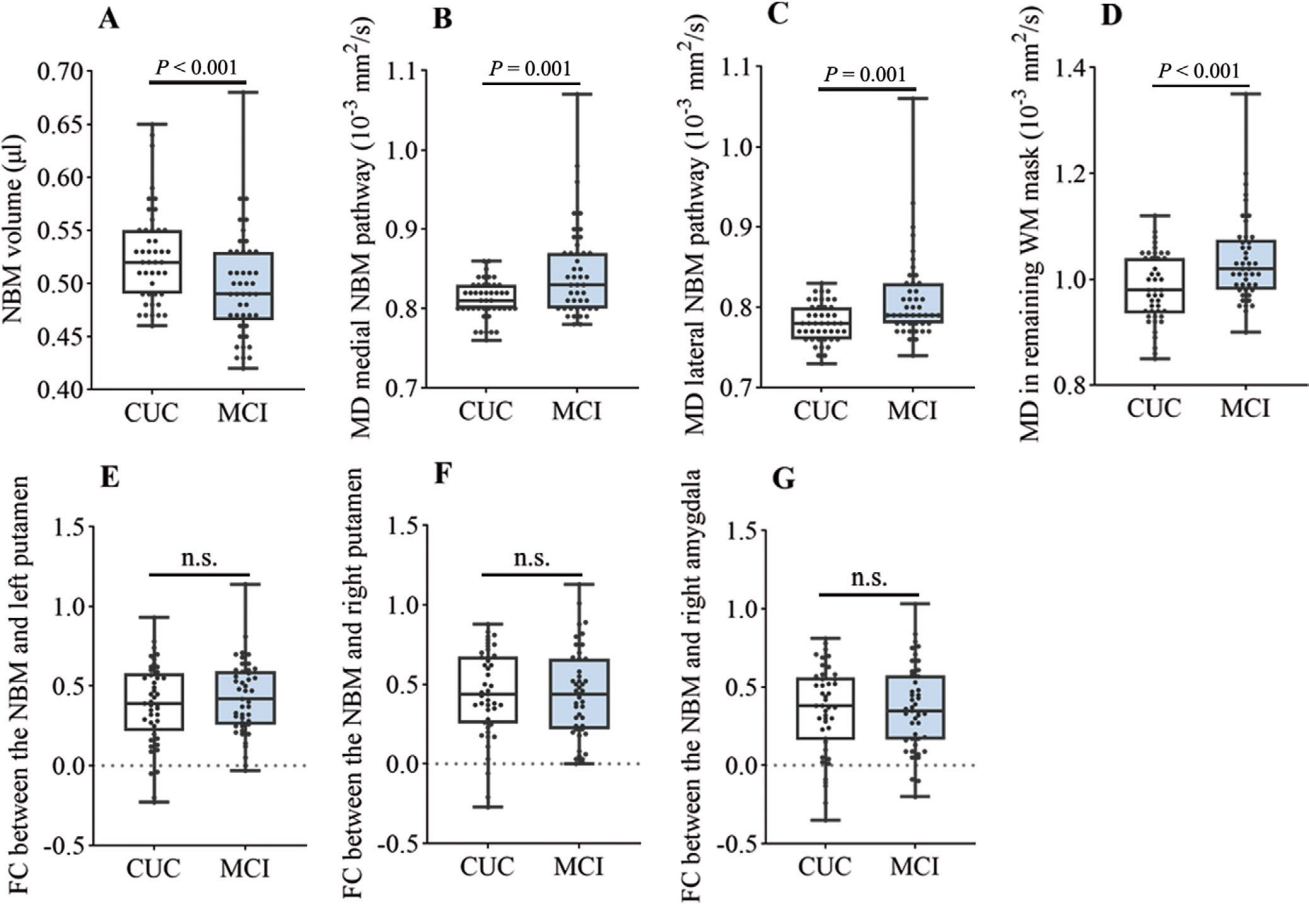
Difference in NBM and related pathways between the MCI and CUC groups. (A) NBM volume was significantly reduced in the MCI group compared to the CUC group. Average MD value was increased in the tracked pathways (B and C) and the remaining WM (D) in the MCI group as compared to the CUC group. (E–G) The functional connections between NBM ROI and three related brain regions did not differ between the two groups (*p* > 0.05). FC, functional connection; MD, mean diffusion; n.s., not significant; NBM, nucleus basalis of Meynert; WM, white matter.

Compared with the CUC group, the MCI group presented a significant reduction in the NBM volume (*F* = 12.717, *p* < 0.001) and increased average MD values in both the tracked cingulum pathway (*F* = 11.594, *p* = 0.001) and the external capsule pathway (*F* = 13.055, *p* = 0.001) as well as in the remaining WM after controlling for age, sex, education, and TIV (Figure 1A–D, Table 1).

There was no significant difference in functional connections between the NBM ROI and three related brain regions (bilateral putamen and right amygdala) after controlling for age, sex, and years of education (Figure 1E–G, Table 1).

### 3.3 Clinical Correlation of NBM Parameters Across All Participants

There were extensive correlations between cognitive scores and NBM volume and NBM‐related pathways (Table 2). The NBM volume was positively correlated with the MMSE (*r* = 0.266, *p* = 0.011), MoCA (*r* = 0.217, *p* = 0.040), and TMT‐A (*r* = 0.225, *p* = 0.033) scores. The MD values in the medial pathway were negatively correlated with the MMSE (*r* = −0.403, *p* < 0.001), MoCA (*r* = −0.366, *p* < 0.001), TMT‐A (*r* = −0.320, *p* = 0.002), and forward DS test (*r* = −0.321, *p* = 0.002) scores. The MD values in the lateral pathway were negatively correlated with the MMSE (*r* = −0.310, *p* = 0.003), MoCA (*r* = −0.319, *p* = 0.002), TMT‐A (*r* = −0.289, *p* = 0.006), and forward DS test (*r* = −0.385, *p* < 0.001) scores. Moreover, the MD values in the remaining WM were negatively correlated with the MoCA (*r* = −0.240, *p* = 0.023), TMT‐A (*r* = −0.359, *p* = 0.001), and forward DS test (*r* = −0.331, *p* = 0.001) scores.

**TABLE 2:** Correlation between cognitive scores and NBM metrics in all participants.

	NBM volume		MD (×10^−3^)						FC					
			Medial NBM pathway		Lateral NBM pathway		Remaining WM		Between NBM and left putamen		Between NBM and right putamen		Between NBM and right amygdala	
	*r*	*p*	*r*	*p*	*r*	*p*	*r*	*p*	*r*	*p*	*r*	*p*	*r*	*p*
MMSE	0.266	0.011	−0.403	< 0.001	−0.310	0.003	−0.196	0.064	0.157	0.140	0.177	0.095	0.020	0.852
MoCA	0.217	0.040	−0.366	< 0.001	−0.319	0.002	−0.240	0.023	0.038	0.720	0.006	0.955	−0.083	0.439
TMT‐A	0.225	0.033	−0.320	0.002	−0.289	0.006	−0.359	0.001	−0.089	0.405	−0.007	0.949	0.111	0.298
Forward DS	−0.051	0.631	−0.321	0.002	−0.385	< 0.001	−0.331	0.001	−0.116	0.277	−0.051	0.632	−0.160	0.131
Backward DS	0.056	0.599	−0.077	0.470	−0.183	0.085	−0.151	0.155	−0.043	0.691	0.050	0.640	−0.023	0.827

*Note:* Spearman's correlations were calculated between neuropsychological scores and MRI metrics.

Abbreviations: DS, Digit Span; FC, functional connection; MD, mean diffusivity; MMSE, Mini‐Mental State Examination; MoCA, Montreal Cognitive Assessment; NBM, nucleus basalis of Meynert; TMT, Trial Making Test; WM, white matter.

In addition, the correlations between different imaging metrics were analyzed. The MD values were significantly correlated between the two tracked cholinergic pathways (*r* = 0.767, *p* < 0.001) and between the remaining WM and both the medial pathway (*r* = 0.456, *p* < 0.001) and the lateral pathway (*r* = 0.701, *p* < 0.001). fMRI revealed that the FCs between the NBM and bilateral putamen were correlated with each other (*r* = 0.734, *p* < 0.001) and were also correlated with the FC between the NBM and right amygdala (left putamen: *r* = 0.248, *p* = 0.018; right putamen: *r* = 0.340, *p* = 0.001). Furthermore, the MD in the remaining WM was negatively correlated with the NBM volume (*r* = −0.286, *p* = 0.006) and the FC between the NBM and right amygdala (*r* = −0.258, *p* = 0.014).

### 3.4 Effects of CCT on Cognitive Function and NBM Parameters in MCI Patients

Among all 45 MCI patients, 36 provided consent to participate in the CCT trial and were subsequently randomized into the CCT group (*n* = 18) or the control group (*n* = 18). Four patients in the CCT group dropped out during the study because they forgot how to use the tablet computers (*n* = 2) or failed to complete the targeted training tasks on time (*n* = 2). Finally, 32 participants (14 in the CCT group and 18 in the control group) reached the training goals and were assessed at the posttraining follow‐up. Because the follow‐up DTI image of one patient in the control group was unqualified, the analysis of DTI data was performed based on 31 participants (Figure S2).

**FIGURE 2:**
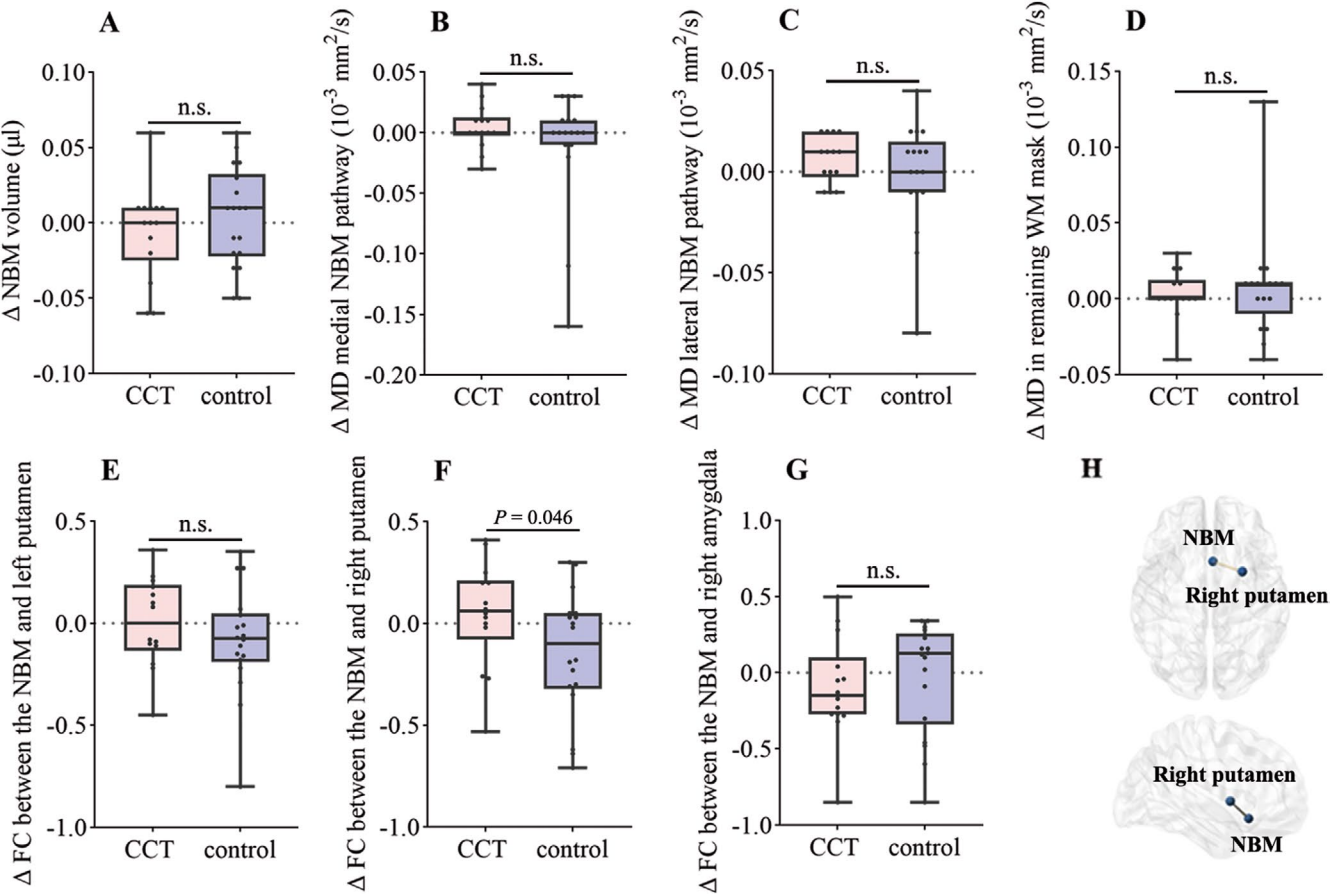
Changes in the NBM parameters. There was significant group × time effect on the FC between NBM and right putamen (F, H). No significant group × time effects were observed in the structural MRI and other fMRI metrics (A–E, G). CCT, computerized cognitive training; FC, functional connection; MD, mean diffusion; n.s., not significant.

For the MCI patients who completed the CCT trial, there were no differences in age (*W* = 132.5, *p* = 0.819), sex (*χ* ^2^ = 1.524, *p* = 0.217) or education (*W* = 121, *p* = 0.858) between the CCT and control groups. The baseline cognitive scores did not significantly differ between these two groups either. Table 3 presents the comparisons of between‐group effects on neuropsychological data and MRI parameters. MMSE (*p* = 0.041) and backward DS test (*p* = 0.004) scores showed significant time effects. A significant group × timepoint effect on the backward DS test score (*p* = 0.021) was observed, but this effect was absent for the scores of the other tests, indicating that the backward DS test score significantly improved in the CCT group than in the control group after 12 weeks of intervention. For MRI metrics, there was a significant group × time effect on the FC between the NBM and right putamen (*p* = 0.046; Table 3, Figure 2H). No significant group × time effects on the NBM volume, the integrity of the two cholinergic pathways, or the FC between the NBM and other related brain regions were detected.

**TABLE 3:** Comparisons of between‐group effects on neuropsychological data and MRI parameters for the training trial.

	CCT group (*n* = 14)			Control group (*n* = 18)			Group	Time	Group × Time
	Baseline	Post	Change	Baseline	Post	Change	*p*	*p*	*p*
MMSE	27.43 (2.21)	26.00 (2.60)	−1.43 (1.95)	26.72 (1.99)	26.00 (3.01)	−0.72 (2.44)	0.327	0.041	0.564
MoCA	22.00 (3.70)	22.57 (3.72)	0.57 (2.03)	22.44 (3.94)	21.83 (4.45)	−0.61 (2.43)	0.744	0.696	0.572
TMT‐A	−52.43 (25.25)	−57.93 (25.49)	−5.50 (15.78)	−64.67 (33.05)	−66.56 (36.57)	−1.89 (21.00)	0.266	0.228	0.284
Forward DS	7.43 (1.22)	7.64 (1.22)	0.21 (0.80)	7.06 (1.43)	6.94 (1.51)	−0.11 (0.90)	0.273	0.836	0.106
Backward DS	4.36 (1.15)	5.21 (0.89)	0.86 (0.86)	4.11 (1.28)	4.33 (1.41)	0.22 (0.73)	0.137	0.004	0.021
NBM volume (μL)	0.505 (0.041)	0.500 (0.037)	−0.005 (0.032)	0.497 (0.043)	0.501 (0.048)	0.004 (0.032)	0.864	0.990	0.122
MD in Medial NBM pathway (×10^−3^)	0.825 (0.034)	0.829 (0.040)	0.005 (0.017)	0.850 (0.077)	0.838 (0.051)	−0.011 (0.048)	1.000	1.000	1.000
MD in Lateral NBM pathway (×10^−3^)	0.793 (0.034)	0.799 (0.040)	0.006 (0.011)	0.808 (0.042)	0.806 (0.030)	−0.002 (0.028)	1.000	1.000	1.000
MD in remaining white matter (×10^−3^)	1.012 (0.064)	1.015 (0.068)	0.003 (0.016)	1.029 (0.057)	1.037 (0.052)	0.007 (0.036)	0.337	0.238	0.552
FC between NBM and left putamen	0.461 (0.200)	0.466 (0.249)	0.005 (0.220)	0.432 (0.277)	0.347 (0.165)	−0.085 (0.264)	0.244	0.319	0.262
FC between NBM and right putamen	0.394 (0.263)	0.439 (0.215)	0.044 (0.259)	0.474 (0.314)	0.332 (0.211)	−0.144 (0.303)	0.847	0.290	0.046
FC between NBM and right amygdala	0.404 (0.227)	0.300 (0.334)	−0.104 (0.333)	0.398 (0.289)	0.378 (0.233)	−0.020 (0.362)	0.627	0.311	0.486

*Note:* The Group × Timepoint interaction effect on the neuropsychological measures and MRI parameters was tested with a linear mixed model (LMM) for repeated measures at baseline and the end of the intervention.

Abbreviations: CCT, computerized cognitive training; FC, functional connection; MD, mean diffusivity; MMSE, Mini‐Mental State Examination; MoCA, Montreal Cognitive Assessment; NBM, nucleus basalis of Meynert; TMT, Trial Making Test.

No study‐related adverse events were reported in either the CCT or control groups.

## 4 Discussion

In this study, we focused on the structural and functional changes in the NBM and related pathways, including NBM volume, DTI‐based integrity of WM pathways, and fMRI‐based functional projections originating from the NBM, in patients with MCI. We detected a reduction in the NBM volume and the disruption of the integrity of both cholinergic tracts, which demonstrated central nervous system cholinergic system impairment in MCI patients. Moreover, 12 weeks of CCT had significant effects on cognitive function, for example, attention and working memory, and functional connections between the NBM and right putamen, suggesting that enhanced NBM‐related neuron activities might contribute to cognitive improvement in MCI patients. To our knowledge, this is the first study to explore the effects of CCT on the NBM via multimodal MRI analysis in patients with MCI.

The NBM projects cholinergic nerves to the cerebral cortex, and degeneration of this region is one of the main pathophysiological processes of AD. A histological study revealed that the loss of cholinergic neurons within the NBM was associated with impaired cholinergic pathways and cholinergic denervation to the frontal and temporal lobes in AD patients [35] and might even occur at the MCI stage [36]. In this study, we observed atrophy of the NBM in MCI patients and disruption of the WM microstructure within the two major NBM cholinergic pathways, consistent with a previous study [8], supporting structural changes in the NBM and associated cholinergic system at early stage of AD. In addition, the integrity of the remaining WM was impaired in patients with MCI and was correlated with the NBM volume, both the medial and lateral NBM pathways, and the NBM FC with the right amygdala, indicating that the degeneration of the NBM and cholinergic system is not specific but occurs concomitantly with the whole‐brain pathological process of AD or other types of dementia.

We did not observe significant alterations in FC between the NBM and three related brain regions (locations of FC maxima for pCBF, i.e., bilateral putamen and right amygdala) in MCI patients. Although previous studies revealed reduced NBM FC with several brain areas, such as the frontal cortex, temporal cortex, cingulate cortex, insula, and claustrum, in MCI patients [10, 11, 37], these studies also revealed no changes in NBM FC with the putamen or amygdala.

Moreover, the integrity of the two NBM WM pathways was correlated with all assessed cognitive domains, such as attention, processing speed, and working memory, while the NBM volume was correlated less strongly with global cognition and attention in all participants, including both MCI patients and CUCs. Previous findings in normal aging also supported that the integrity of NBM WM pathways might make a stronger contribution to cognition than the NBM volume [38]. Both the lateral pathway and the medial pathway project to cortices (e.g., frontal cortex, and cingulate cortex, respectively) related to processing speed. In addition, the lateral pathway projects to cortices in the frontal lobe and posterior cortex, which are involved in attention [8]. Our findings suggest that the degeneration of the cholinergic system is associated with a broad cognitive decline at the early stage of AD or related dementia. The participants with MCI showed significant improvement in the backward DS test score after receiving 12 weeks of adaptive cognitive training. The backward DS test reflects working memory ability since it requires simultaneous storage and processing while exerting attentional control [39]. This result is consistent with the findings from a meta‐analysis of 12 studies that showed moderate and significant effects of cognitive training on working memory and attention in MCI patients [40].

Moreover, functional connectivity between the NBM and right putamen was increased by the CCT intervention, which supported neuroplasticity of even aging or diseased brains and the application of cognitive training in MCI patients. Asymmetric disruption of the NBM FC was found in MCI patients in a recent study, with decreased FC between the right middle temporal gyrus, right superior frontal gyrus and right anterior cingulate and the right NBM but only between the left inferior frontal gyrus and the left NBM when analyzing the bilateral NBM and bilateral hemisphere regions separately [11]. Another study measuring cortical FC profiles reported reduced FC of the pCBF with the right midcingulate and right superior temporal area but not the left cortex in MCI patients compared with that in CUCs [37]. These findings indicated that FC of the NBM with the right brain hemisphere might be more susceptible to MCI; in particular, FC with the right putamen is sensitive to cognitive training. Similarly, a multimodal MRI‐based study revealed a disruption of FC between the NBM and the right rather than the bilateral insula in AD patients [41]. Furthermore, the asymmetric change in cholinergic connections was supported by autopsy findings, which suggested that more AD patients had a lower density of muscarinic receptors in the right than in the left medial hippocampal cortex [42]. However, another study based on the postmortem brain of AD patients indicated that defects in cholinergic activity tended to occur bilaterally [43]. Therefore, the association between cholinergic changes in either hemisphere and cognitive impairment still needs further investigation.

This study investigated features of NBM structural and functional projections using multimodal MRI data and explored their changes after cognitive training in MCI patients. There are several limitations in the present study. First, the MCI patients included in this study might have had diverse etiologies, not just AD, although we attempted to exclude patients with other diseases or conditions causing cognitive impairment via a comprehensive medical history and physical, laboratory, and neuroimaging evaluations. Therefore, our findings need to be validated in a larger cohort of patients with MCI that is due to AD, with their diagnosis supported by core AD biomarkers. Second, except for attention, processing speed, and working memory, other cognitive domains, such as language and visuospatial function, were not evaluated and analyzed in the present study. The contributions of the NBM pathways to cognitive function need more comprehensive investigations. Third, we chose only three locations as the FC maxima for the pCBF based on a previous study and disregarded the remaining FC profile of the pCBF. Although the pCBF covers most of the NBM and the FC profile of the pCBF largely replicates the FC profile of the NBM, the anterior‐medial part of the NBM, which is part of the aCBF, was not analyzed in this study. As a result, we may have underestimated the effect of cognitive training on FC projections in the NBM. Finally, the small sample size of participants in the CCT trial could have lowered the power of the analyses, causing an underestimation of the effects of CCT on neuropsychological test scores and NBM‐related MRI parameters. Furthermore, structural changes may require a longer observation period, and the maintenance effect after cognitive training remains to be explored at long‐term follow‐ups in future studies.

## 5 Conclusion

This study demonstrated that degeneration of the NBM accompanied by a reduction in the integrity of its WM pathways occurred in MCI patients and was correlated with cognitive impairment. Furthermore, working memory and FC between the NBM and right putamen could be improved by computerized, multidomain, and adaptive cognitive training.

## Author Contributions

Qingzheng Lu drafted the manuscript and performed statistical analysis. Yu Wang processed and analyzed images. Bingqian Qu, Qingzheng Lu, Caixia Wang, Xiao Su, and Siqi Wang evaluated the participants. Bingqian Qu and Qingzheng Lu performed the neuropsychological assessment. Yi Tang and Yi Xing contributed to the study design. Wen Qin conducted the MRI procedure and acquired the imaging data. Nan Zhang designed the study, recruited participants, and substantially revised the manuscript. All authors read and approved the final version of the manuscript.

## Conflicts of Interest

The authors declare no conflicts of interest.

## Supporting information


Data S1.

## Data Availability

The data that support the findings of this study are available from the corresponding author upon reasonable request.
