# Supplementary material for: Structural and Functional Projections of the Nucleus Basalis of Meynert and Their Changes After Cognitive Training in Individuals With Mild Cognitive Impairment
Source: CNS Neurosci Ther. 2024 Dec 26;30(12):e70194. doi: 10.1111/cns.70194 (PMC11669844; doi:10.1111/cns.70194)
Supplement: Supplementary file 1 — Data S1. [file CNS-30-e70194-s001.docx]

**Supplemental Materials**

**Structural and functional projections of the nucleus basalis of Meynert and their changes after cognitive training in individuals with mild cognitive impairment**

Qingzheng Lu^1^, Yu Wang^1^, Bingqian Qu^2^, Caixia Wang^1,3^, Xiao Su^1^, Siqi Wang^1^, Yi Xing^4^, Wen Qin^5^, Yi Tang^4^, Nan Zhang^1,2^

1 Department of Neurology, Tianjin Neurological Institute, Tianjin Medical University General Hospital, Tianjin, China.

2 Department of Neurology, Tianjin Medical University General Hospital Airport Site, Tianjin, China.

3 Department of Neurology, Baotou Central Hospital, Baotou, China.

4 Department of Neurology & Innovation Center for Neurological Disorders, Xuanwu Hospital, Capital Medical University, National Center for Neurological Disorders, Beijing, China.

5 Department of Radiology and Tianjin Key Laboratory of Functional Imaging,

Tianjin Medical University General Hospital, Tianjin, China.

**List of Supplemental Materials**

1. Figure S1: Pipeline of NBM white matter tract estimation.
2. Figure S2: Flowchart for the CCT study.

**
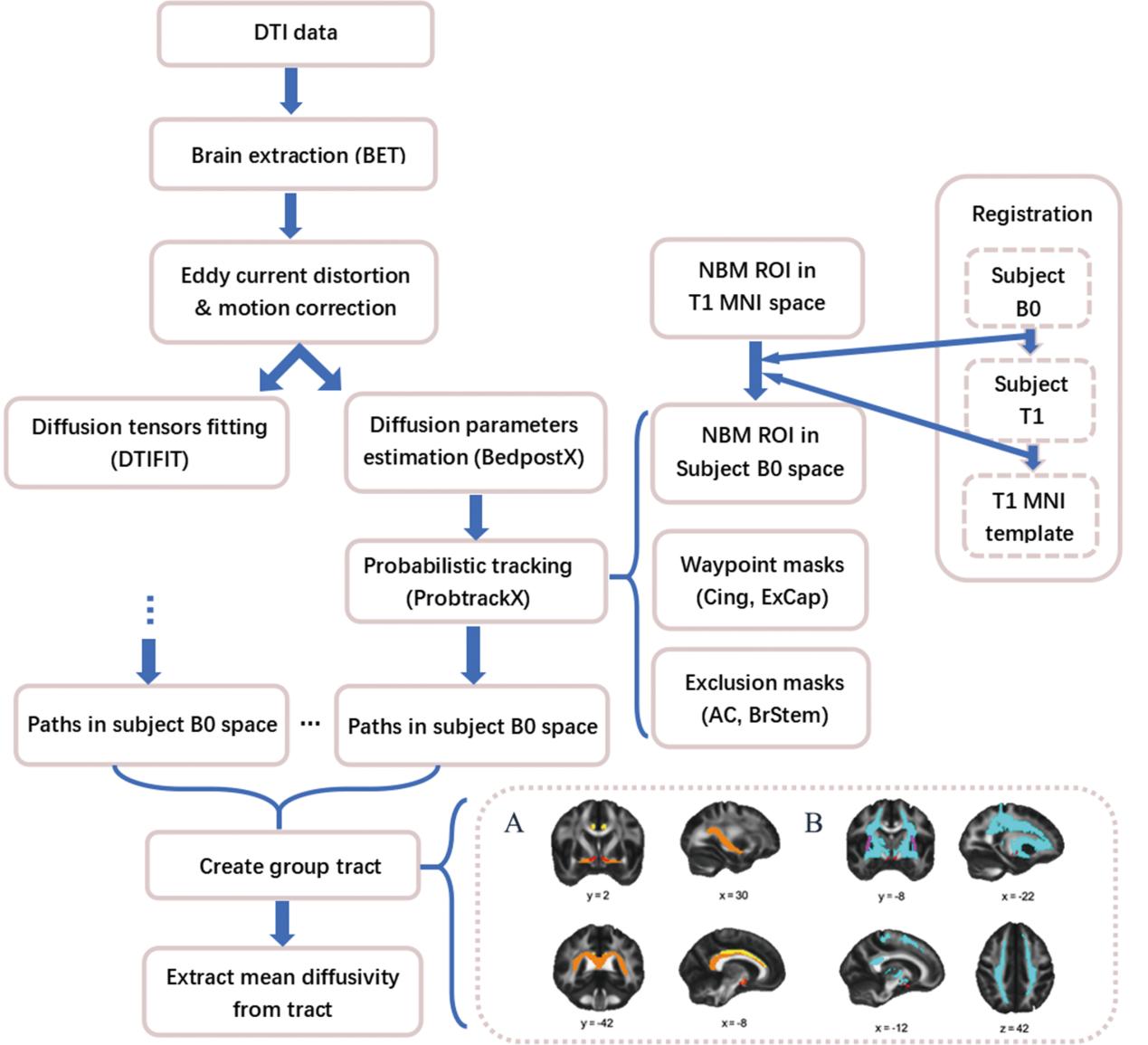
**

**Figure S1** **Pipeline of NBM white matter tract estimation.** (A) Medial NBM pathway (shown in orange) estimated with the NBM region of interest as seed (shown in red), the cingulum as waypoint mask (shown in yellow), and brainstem and anterior commissure as exclusion masks. (B) Lateral NBM pathway (shown in cyan) estimated with the NBM region of interest as seed (shown in red), the external capsule as waypoint mask (shown in pink), and the brainstem and anterior commissure as exclusion masks. DTI, diffusion tensor imaging; NBM, nucleus basalis of Meynert; ROI, region of interest; MNI, Montreal Neurological Institute; AC, anterior commissure; BrStem, brain stem. B0 images (DTI b = 0).


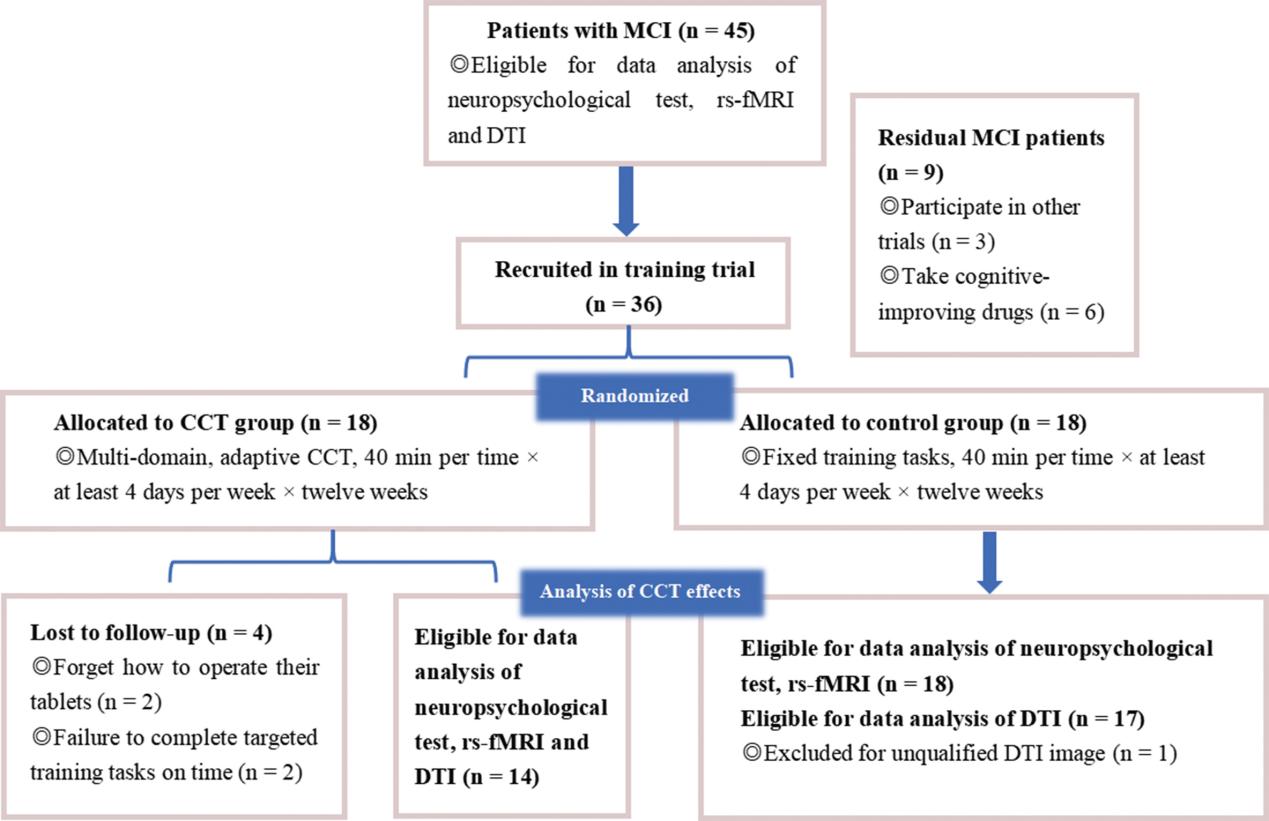


**Figure S2** **Flowchart for the CCT study.** MCI, mild cognitive impairment; rs-fMRI, resting-state functional MRI; DTI, diffusion tensor imaging; CCT, computerized cognitive training.
